# Hypokalemic Periodic Paralysis: A Rare Case of a Descending Flaccid Paralysis

**DOI:** 10.7759/cureus.55981

**Published:** 2024-03-11

**Authors:** Inder Preet Singh Bhatia, Jayaraj Hasvi, Pandaramparambil Saidu Nazneen, Amit Rajan

**Affiliations:** 1 Department of Internal Medicine, 167 Military Hospital, Pathankot, IND; 2 Department of Internal Medicine, Divisional Railway Hospital, Tiruchirappalli, IND; 3 Department of Lab Sciences and Pathology, 167 Military Hospital, Pathankot, IND

**Keywords:** weakness, skeletal muscles, potassium, flaccid paralysis, hypokalemia

## Abstract

Hypokalemic periodic paralysis (HPP) is an uncommon condition resulting from channelopathy, impacting skeletal muscles. It is distinguished by episodes of sudden and temporary muscle weakness alongside low potassium levels. The normalization of potassium resolves the associated paralysis. Most of these cases are hereditary. Few cases are acquired and are associated with an etiology related to endocrine disorders (e.g., thyrotoxicosis, hyperaldosteronism, and hypercortisolism). It is characterized by acute flaccid paralysis, usually of the ascending type, affecting the proximal region more than the distal region. Herein, we report the case of a 29-year-old male who instead of the ascending type presented with descending-type acute flaccid paralysis. Potassium level at presentation was 1.7 mEq/L. The patient was managed with parenteral and oral potassium supplementation, after which the weakness was completely resolved.

## Introduction

Hypokalemic periodic paralysis (HPP) is a rare disorder, with an estimated prevalence of one in 100,000 individuals [1]. This condition arises from channelopathy, primarily impacting skeletal muscles. It is marked by episodes of sudden and reversible muscle weakness accompanied by hypokalemia [1]. The bulbar and respiratory muscles are usually spared; however, their involvement can occur in cases of severe hypokalemia [2]. Normalization of potassium results in complete neurological recovery. Most cases are hereditary, and a few of them are acquired and are attributed to the etiology of endocrine disorders (thyrotoxicosis, hyperaldosteronism, and hypercortisolism) [2]. It is characterized by acute flaccid paralysis, usually of the ascending type, affecting the proximal muscles more than the distal ones [1]. Various triggers have been established, such as strenuous physical activity, vomiting, a carbohydrate-rich diet, and exposure to cold environments.

## Case presentation

A 29-year-old male patient experienced two episodes of vomiting after running cross country. The patient reported symptoms of excessive hunger, dry mouth, sweating, and nervousness. Over the next six hours, the individual had difficulty in lifting his neck, followed by weakness in the bilateral upper limbs, truncal region, and involvement of lower limb muscles over the next six to eight hours. Weakness was associated with a loosening of limbs. The weakness was symmetrical in nature, with involvement of the proximal muscles more than that of the distal muscles. There was no history of paresthesia/loss of hot or cold sensation. No history of bowel or bladder control issues. The patient had no history of snakebites. The patient had no history of diuretic or glucocorticoid use. He had a similar history in the past six months, for which he was admitted to a different hospital, and there was a complete resolution of symptoms after five to six days. The patient had no relevant family histories.

Examination revealed a power of 0/5 in the neck region, 2/5 at the shoulder level, 4-/5 at the elbow level, 4+/5 at the wrist level, 2/5 at the hip level, 4-/5 at the knee level, and 4+/5 at the ankle level. Deep tendon reflexes were preserved. The sensory examination results were normal. Investigations revealed a K+ of 1.7 mEq/L. The magnesium level at presentation was 1.94 mg/dL. The rest of the hematological and biochemical parameters were normal. Arterial blood gas analysis showed pH of 7.47, pCO_2_ of 31 mmHg, and HCO_3_ of 21.6 mmol/L. ECG showed U waves with prolonged QTc intervals. Free T3 was 3.40 pg/ml (2.0-4.0), free T4 was 1.61 ng/dl (0.93-1.70), and TSH was 1.1.56 uIU/ml (0.27-4.20). Serum aldosterone was 4.20 ng/dl, and renin was 12.01 mIU/L with an aldosterone/renin ratio of 3.497 (normal, <20.60). Serum cortisol was 22.0 ug/dl (4.30-22.40), as shown in Table 1. No history and examination suggested facial and skeletal anomalies, so Andersen-Tawil syndrome (ATS) was not considered.

**Table 1:** Investigative profile

Na+ (137 – 145 mEq/l)- 136	K+(3.6 – 5.0 mEq/l)- 1.7	Calcium (8.4- 8.510.2mg/dl)- 9.2
Urea – 19 mg/dl	Creatinine (0.8-1.5 mg/dl)- 0.9	S. cortisol (4.30-22.40 ug/dl) – 22.87
Free T3 (2.0-4.40 pg/ml) – 3.40	Free T4 (0.93-1.70 ng/dl) – 1.61	TSH (0.27-4.20 uIU/ml) – 1.56
S. aldosterone (1.17-23.60 ng/dl)- 4.20	S. renin (2.80-39.90 mIU/l – 12.01	Aldosterone/renin ratio (<20.60 ng/mIU) – 3.49
MRI brain – no significant abnormality		

He was managed for HPP with oral and parenteral potassium supplementation. Serum potassium levels normalized after three days of supplementation. Over the next three to four days, the weakness was completely resolved. The individual was offered genetic testing but was unwilling to do so. The patient had no symptoms on follow-up at one and two months after discharge.

## Discussion

HPP is more prevalent in Asian populations and typically affects males between the ages of 20 and 40 years. Males are more commonly affected than females [3]. The movement of potassium into the intracellular fluid (ICF) is facilitated by the Na+/K+/ATPase pump, which also transports sodium to the extracellular fluid (ECF). This pump is present in various tissues, such as the liver, muscles, and kidneys. The outward movement of potassium is regulated by inward-rectifying K channels [4]. These channels work together to tightly regulate potassium levels. Hormones play a role in modulating the activity of this pump.

Thyrotoxic periodic paralysis, although rare, can manifest as symmetrical weakness in the lower limbs initially, potentially progressing to affect all limbs and respiratory muscles. However, our patient's normal thyroid panel eliminated this possibility. Another uncommon genetic condition, ATS, characterized by paralysis, cardiac arrhythmias, and specific skeletal and facial features, was also ruled out as the patient lacked skeletal anomalies consistent with ATS. It is crucial to investigate other metabolic imbalances to exclude secondary causes of hypokalemia, such as gastrointestinal, renal, endocrine, or iatrogenic issues. Our patient's urinary potassium level of 4.39 mEq/L, no evidence of metabolic acidosis, and normal serum aldosterone levels suggested against intrarenal potassium wasting disorders, such as renal tubular acidosis or increased aldosterone activity. Normal serum magnesium levels further discounted magnesium deficiency as a cause. In addition, normal plasma renin activity and color Doppler of the renal vessels ruled out renin-secreting tumours and renal artery stenosis. A thorough medical history and physical examination helped rule out the influence of diuretics, dietary factors, or gastrointestinal problems [4].

Catecholamines can also stimulate the Na+/K+/ATPasepump activity through β-adrenergic receptors. Insulin and testosterone have been observed to enhance the activity of the Na-K pump, while estrogen may have the opposite effect, potentially explaining the higher incidence of HPP in males.

Certain genetic factors, combined with triggers, such as thyrotoxicosis, contribute to the clinical presentation of HPP. Inward rectifying K+ channels (Kir) act in opposition to the Na+/K+/ATPase pump by facilitating the extracellular movement of K+. KCNJ2 and KCNJ18 are the predominant genes encoding Kir channels in Asians and Caucasians, respectively. Mutations in genes, such as KCNJ, CACNA, and SCN4A, as well as elevated levels of thyroid hormones, catecholamines, or insulin, impede the efflux of potassium from cells, leading to potassium sequestration and the development of hypokalemia [3,5-7].

The clinical manifestation of HPP ranges from mild, temporary motor dysfunction to severe cases involving complete flaccid paralysis, which may include the respiratory muscles, particularly in severe instances. HPP may precipitate arrhythmias, such as ventricular tachycardia, ventricular fibrillation, and AV block, mainly due to hypokalemia [4].

Paralytic episodes in HPP can be triggered by various factors, including heavy meals, alcohol consumption, physical exercise, a diet high in salt, stress, infections, and the use of glucocorticoids [4]. In our case, the precipitating factor was likely strenuous physical activity.

Potassium must be supplemented cautiously as in cases of relative hypokalemia, potassium is shifted into the cells rather than being depleted from the body, and rebound hyperkalemia can occur secondary to overcorrection [8]. In addition, we considered the correction of associated electrolytes, such as magnesium, when repleting for potassium. Various non-pharmacological and pharmacological methods have been developed to prevent future attacks. Among non-pharmacological, patients should be educated to avoid triggers and dietary modifications to avoid large-carbohydrate meals.

The pharmacological therapies include carbonic anhydrase inhibitors and potassium-sparing diuretics. Regular monitoring of renal function should be performed when patients are being treated with these drugs [4]. Recurrent attacks of hypokalemic paralysis can lead to frequent hospitalizations, which can affect the patient’s personal and professional lives.

Our patient was managed with carbonic anhydrase inhibitors and potassium-sparing diuretics and had favorable outcomes, highlighting the importance of correcting hypokalemia.

## Conclusions

HPP is a rare cause of acute flaccid paralysis usually of ascending in nature. Weakness usually involves more proximal muscles than distal ones. The diagnosis of our case was confirmed by hypokalemia during the attack. This condition should not be overlooked because it is reversible. There is a complete recovery of neurological deficits after correction of potassium; however, care should be taken not to overcorrect the potassium level, as this may lead to hyperkalemia. Suspicions should be high in cases with a previous personal history or similar family history. Attempts to identify the triggers should be made, as prevention of these triggers can decrease the frequency of attacks and recurrent hospitalization. Patients should be counseled for genetic testing; however, this may not always reveal mutations, as many mutations are still unknown.
